# Associations of serum indolepropionic acid, a gut microbiota metabolite, with type 2 diabetes and low-grade inflammation in high-risk individuals

**DOI:** 10.1038/s41387-018-0046-9

**Published:** 2018-05-25

**Authors:** Marjo Tuomainen, Jaana Lindström, Marko Lehtonen, Seppo Auriola, Jussi Pihlajamäki, Markku Peltonen, Jaakko Tuomilehto, Matti Uusitupa, Vanessa D. de Mello, Kati Hanhineva

**Affiliations:** 1 Department of Clinical Nutrition, Institute of Public Health and Clinical Nutrition, University of Eastern Finland, Kuopio, Finland; 2 Department of Chronic Disease Prevention, National Institute for Health and Welfare, Helsinki, Finland; 3 School of Pharmacy, University of Eastern Finland, Kuopio, Finland; 4 LC-MS Metabolomics Center, Biocenter Kuopio, Kuopio, Finland; 5 Clinical Nutrition and Obesity Center, Kuopio University Hospital, Kuopio, Finland; 6 Dasman Diabetes Institute, Dasman, Kuwait

## Abstract

We recently reported using non-targeted metabolic profiling that serum indolepropionic acid (IPA), a microbial metabolite of tryptophan, was associated with a lower likelihood of developing type 2 diabetes (T2D). In the present study, we established a targeted quantitative method using liquid chromatography with mass spectrometric detection (HPLC-QQQ-MS/MS) and measured the serum concentrations of IPA in all the participants from the Finnish Diabetes Prevention Study (DPS), who had fasting serum samples available from the 1-year study follow-up (*n* = 209 lifestyle intervention and *n* = 206 control group). Higher IPA at 1-year study was inversely associated with the incidence of T2D (OR [CI]: 0.86 [0.73–0.99], *P* = 0.04) and tended to be directly associated with insulin secretion (*β* = 0.10, *P* = 0.06) during the mean 7-year follow-up. Moreover, IPA correlated positively with dietary fiber intake (g/day: *r* = 0.24, *P* = 1 × 10^−6^) and negatively with hsCRP concentrations at both sampling (*r* = − 0.22, *P* = 0.0001) and study follow-up (*β* = − 0.19, *P* = 0.001). Thus, we suggest that the putative effect of IPA on lowering T2D risk might be mediated by the interplay between dietary fiber intake and inflammation or by direct effect of IPA on β-cell function.

## Introduction

Well-established lifestyle, and metabolic and genetic factors are currently used for stratifying people at high risk of developing type 2 diabetes (T2D). Even though physical inactivity, overweight, and obesity are generally accepted major risk factors contributing to the T2D incidence^1^, the quality of the diet seems also to have a role.

We recently performed a non-targeted metabolite profiling study in pre-selected participants with impaired glucose tolerance (IGT) from the Finnish Diabetes Prevention Study (DPS) and reported that serum indolepropionic acid (IPA) was associated with a lower likelihood of developing T2D^2^. Furthermore, we replicated this association in two other independent cohorts^2^. In DPS, IPA was the only metabolite linked with preservation of β-cell function in those who did not develop T2D^2^.

IPA is a specific microbial product from dietary tryptophan absorbed from the gut into the blood stream and is also found in cerebrospinal fluid^3,4^. In animal models, IPA exerts antioxidant and anti-inflammatory effects and possibly ameliorates glucose metabolism^5,6^.

Because of the putative link of gut microbiota and T2D^7^, we aimed at getting a more accurate picture of the interplay between IPA, T2D, glucose metabolism, inflammation, and diet. Therefore, we established a targeted quantitative method using liquid chromatography with triple quadrupole mass spectrometric detection (HPLC-QQQ-MS/MS) to measure the precise concentrations of IPA in serum samples from the DPS study.

## Research design and methods

### Study participants

The DPS was a randomized, controlled, multicenter study carried out in Finland between the years 1993 and 2001 (ClinicalTrials.gov NCT00518167). A total of 522 individuals with IGT were randomly allocated into either a lifestyle intervention or control group. After a mean 4-year intervention (active study) period, the post-intervention follow-up was carried out with annual examinations. The DPS study design and methods have been reported in detail elsewhere^8^ and are described in the Supplementary Information (SI) material.

The present study included all the participants who had fasting serum samples available for IPA analysis from the one-year follow-up. Altogether, IPA was measured in serum of 415 participants (*n* = 209 lifestyle and *n* = 206 control groups, respectively).

### Laboratory determinations and genotyping

Glucose and insulin levels were determined as previously described^9^ and as surrogate index of the first/early-phase insulin secretion we used the disposition index_30_ (DI_30_)^9^ (details in SI material). High sensitive C-reactive protein (hsCRP) was measured in fasting serum at IPA sampling (1-year follow-up) and yearly during the mean 4-year intervention (active study) period using an IMMULITE® 2000 Systems Analyzer (Siemens Healthcare Diagnostics, Inc. Tarrytown, NY)^2^. Genotyping of *TCF7L2* rs7903146 and rs12255372 was performed as reported^10^.

### Quantitation with HPLC-MS/MS

IPA was quantified by HPLC-QQQ-MS/MS using reversed-phase separation technique. Commercial IPA and IPA-d2 were used as a standard and internal standard, respectively. Details on materials, sample preparation, HPLC-QQQ-MS/MS system, and method validation are described in the SI material.

### Statistical analyses

The data were analyzed using IBM SPSS Statistics 23 software (IBM, Inc., Armonk, NY). After data normalization, Cox proportional hazards regression models assessed the association of IPA with the risk of incident T2D during a mean follow-up of 7 years (range 1–14 years) after IPA sampling. In addition, analysis of variance models adjusted for study group tested the associations of IPA with *TCF7L2* genotypes and with insulin secretion (DI_30_) during the long-term follow-up. For testing correlations, we applied Pearson’s correlation test. A value of *P* < 0.05 was considered significant.

## Results

### IPA concentration and diabetes incidence

After quality control (detailed in SI material), 403 samples (202 lifestyle intervention and 201 control) were included in the final analyses (Table 1). IPA concentration was not different between the study groups (*P* = 0.14, Table 1). When participants with incident T2D at IPA sampling (1-year examination) were excluded (*n* = 4, intervention and *n* = 13, control), the results remained the same (*P* = 0.12).

**Table 1:** Characteristics of the participants at serum IPA sampling (1-year examination study) (*n* = 403)

	Intervention (202)	Control (201)	*P* ^*^
Age (years)	56.2 ± 7.0	55.0 ± 7.0	0.11
Sex (male/female)	70/132	58/143	0.24
Body weight (kg)	82.0 ± 13.4	84.7 ± 14.5	0.05
BMI (kg/m^2^)	29.6 ± 4.3	30.8 ± 4.6	0.006
Plasma glucose (mmol/l)			
Fasting	5.9 ± 0.7	6.2 ± 0.9	0.0001
120 min	8.1 ± 1.9	8.5 ± 2.1	0.01
Serum insulin (pmol/l)			
Fasting	76 (63; 104) (196)	90 (63; 118) (193)	0.02
120 min	358 (236; 606) (190)	438 (313; 705) (189)	0.02
IPA (ng/ml)	192 (118; 291)	174 (104; 276)	0.14

*ANOVA* analysis of variance, *BMI* body mass index, *IPA* indolepropionic acid

Data are mean ± SD, median (interquartile range) or (*n*). *P for the difference between groups at 1-year study using one-way ANOVA for continuous variables or Fisher’s exact test for sex variables.

During a mean follow-up of 7 years since IPA sampling, the number of diabetes cases was 95 in the control and 71 in the intervention groups. Participants who progressed from IGT to diabetes compared with those who did not had reduced levels of IPA at 1-year follow-up (169 [104–264] vs. 207 [118–304] ng/ml, respectively; *P* = 0.05).

We observed that higher IPA concentrations were inversely associated with the incidence of diabetes during the mean 7-year follow-up (odds ratio [confidence interval]: 0.86 [0.73–0.99], *P* = 0.04). A 1 SD increase in IPA was associated with a 14% decrease in the risk of developing diabetes. However, the association lost its significance when body mass index (BMI) (*P* = 0.15), fasting (*P* = 0.07), or 2 h (*P* = 0.26) plasma glucose at IPA sampling were also taken into account. In a model where DPS study group, age and sex were included the association of IPA with diabetes incidence was boderline (*P* = 0.09).

### IPA and insulin secretion

**Fig. 1:**
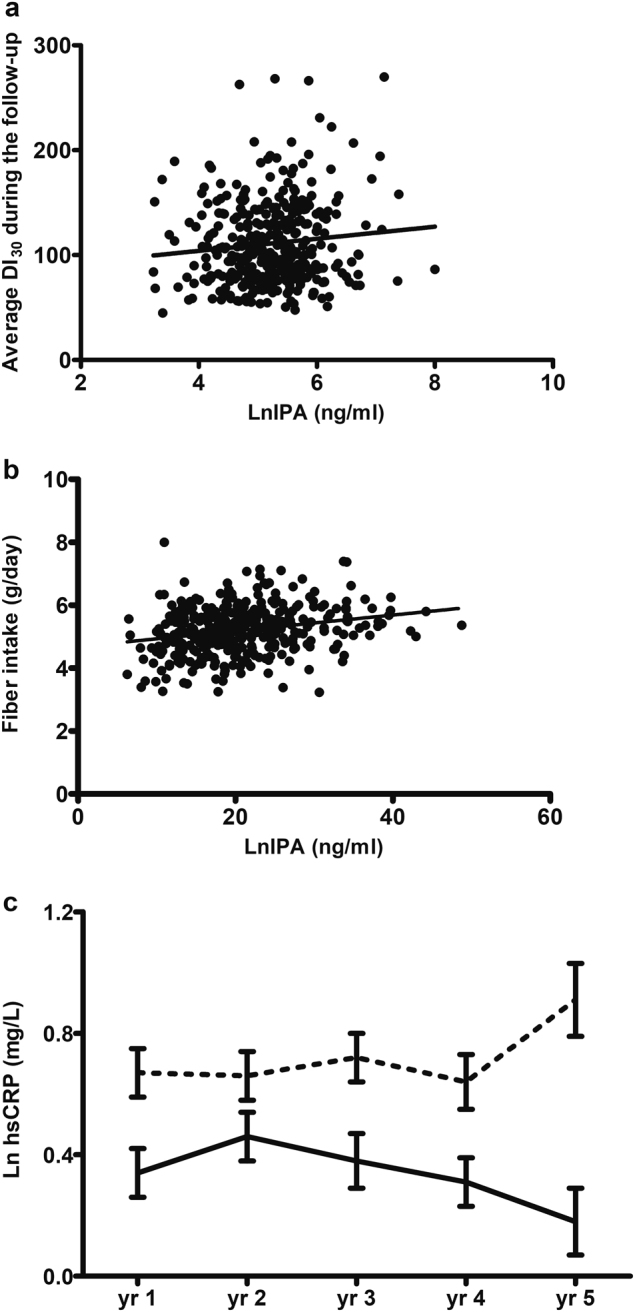
Scattered plot of the relationship of IPA concentrations (natural log transformed; ng/ml) measured from 1-year examination samples and **a**. average insulin secretion (DI_30_) during the mean seven years of follow-up in the DPS (*β* = 0.10, *P* = 0.06) and **b**. fiber intake (g/day) at IPA sampling (*r* = 0.24, *P* = 1 × 10^−6^). **c**. Association of indolepropionic acid (IPA) and hsCRP serum concentrations in DPS (*n* = 291). Descriptive figure of the course of serum hsCRP (natural log transformed; mg/L) during the active study follow-up since IPA sampling (yr 1) according to the median cut-off point in IPA. Solid line = above median cut-off; broken lines = below median cut-off. P = 0.008 for the difference between cut-off point groups

The IPA concentrations tended to be directly associated with insulin secretion (DI_30_) during the mean 7-year follow-up (*β* = 0.10, *P* = 0.06; Fig. 1a). Models adjusted for sex, age, and DPS group retrieved similar results (*P* = 0.07 for the effect of IPA on DI_30_ during the follow-up).

### IPA and *TCF7L2*

Because of the strong relationship of *TCF7L2* genotype with T2D and insulin secretion^10,11^, we tested whether specific related genotypes could interfere in the relationship of IPA with insulin secretion. Overall, these genotypes did not influence IPA concentrations (STable 1) or its association with DI_30_ (*P* > 0.30 for each variant at each respective model).

### IPA correlates with dietary fiber and low-grade inflammation

Our previous results suggested a correlation between IPA and dietary fiber^2^, which was confirmed in the current study (Fig. 1b. *r* = 0.24, *P* = 1 × 10^−6^). There was only a mild correlation between saturated fat intake and IPA, which was no longer significant after controlling for fiber intake (STable 2).

We found a negative correlation of IPA and serum hsCRP levels (*r* = − 0.22, *P* = 0.0001), even after controlling for study group (*P* = 0.0002) or BMI (*P* = 0.001). One-year serum hsCRP was inversely associated with DI_30_ during the long-term follow-up independently of study group (*β* = − 0.14, *P* = 0.01), but not after controlling for BMI (*P* = 0.30).

Serum hsCRP also correlated negatively with fiber intake (*r* = − 0.22, *P* = 6.6 × 10^−5^) after controlling for study group (*P* = 6.6 × 10^−5^) or BMI (*P* = 0.001). When controlled for fiber intake, the correlation between serum IPA and hsCRP concentrations remained significant (*P* = 0.003). We also observed an impact of serum IPA at 1-year study on the average of circulating levels of hsCRP during the 4 years of the study (*β* = − 0.19, *P* = 0.001), independently of the study group (Fig. 1c).

## Discussion

We established the quantitative HPLC-QQQ-MS/MS method for measuring serum IPA in the Finnish DPS. We showed a relationship of IPA with the incidence of T2D using the original design of the DPS and demonstrated a trend for an association with insulin secretion during a longer follow-up time of 7 years. Moreover, we demonstrated that higher serum IPA was associated with lower low-grade inflammation and higher dietary fiber intake. In our study, the predictive value of IPA on T2D incidence weakened after controlling for confounding factors. This suggests that healthy lifestyle changes resulting in higher fiber intake that protected from T2D may modify IPA concentrations and consequently diminish any associations in the whole cohort.

IPA levels correlated negatively with hsCRP concentrations, which has been previously linked with an increased risk of T2D in the DPS population^12^. The gut microbiota seem to have a role in T2D^7^. Therefore, the beneficial effect of increasing dietary fiber and concomitant weight loss on gut microbiota could be linked to the production of IPA, which by enhancing intestinal barrier integrity and lowering inflammation^13–15^, ultimately leads to improved insulin secretion as seen in our study, thereby lowering the risk of T2D. In addition, due to IPA modulation of incretin hormones^5^ this could lead to enhanced insulin secretion.

It has been suggested that higher IPA could ameliorate inflammation^16^ and cell oxidative damage^17,18^, thereby resulting on better insulin secretion due to preservation of β-cells, and consequently lowering the risk of T2D. Accordingly, lower hsCRP concentrations at IPA sampling was associated with a better insulin secretion during the follow-up years. However, it is known that weight loss has an impact on ameliorating beta-cell function and lowering inflammation^9,19,20^. Therefore, it is not surprising that in our study obesity modified the association of insulin secretion with both IPA and inflammation.

Importantly, neither the strongest common T2D-associated variant of *TCF7L2* rs7903146^11^ nor the previously reported T2D-associated variant rs7903146 in DPS^10^ modified the association of IPA levels and insulin secretion, confirming that the effect of *TCF7L2* is probably not mediated by IPA.

Strengths of the present study include the well-characterized and homogenous study population and yearly measurements of insulin secretion estimates during a long follow-up. Moreover, we developed a method for quantification of IPA in serum using all samples available from the DPS. Our study has limitations. The surrogate for insulin secretion (DI_30_) was based on indexes that were not measured by either the hyperinsulinemic-euglycemic clamp or the intravenous glucose tolerance test (IVGTT). Instead, we used an IVGTT for validation^9^.

In conclusion, we propose that the putative beneficial effects of IPA on lowering T2D risk relate to the interplay between high dietary fiber intake and decreased inflammation, or by the direct effect of IPA on β-cell function. Overall, our study further highlights the importance of the gut microbiota as a mediator for the development of metabolic disorders like T2D.

## Electronic supplementary material

Online supplementary information
