# Supplementary material for: Associations of serum indolepropionic acid, a gut microbiota metabolite, with type 2 diabetes and low-grade inflammation in high-risk individuals
Source: Nutr Diabetes. 2018 May 25;8:35. doi: 10.1038/s41387-018-0046-9 (PMC5968030; doi:10.1038/s41387-018-0046-9)
Supplement: Supplementary file 1 — Online supplementary information [file 41387_2018_46_MOESM1_ESM.docx]

**Supplementary information on material, methods and results**

**1. Material and Methods**

**1.1. Diabetes Prevention Study (DPS) original design**

The main inclusion criteria in the DPS were as follows: BMI>25 kg/m2, age 40-64 years, and impaired glucose tolerance (IGT) based on the mean values of two 75 g of glucose oral glucose tolerance tests (OGTT) based on the WHO 1985 criteria. People with previous diagnosis of type 2 diabetes (T2D), severe chronic disease or unstable clinical conditions related to glucose metabolism were excluded from the study. The study protocol was approved by the Ethics Committee of the National Public Health Institute of Helsinki, Finland, and all of the study individuals gave written informed consent.

The individuals in the intervention group received dietary tailored advice aiming at reducing weight and the intake of total and saturated fat and increasing the intake of dietary fiber, and instructions to increase their levels of physical activity. The main goals of the intervention group were weight reduction ≥ 5 %, moderate intensity physical activity ≥ 30 min/d, dietary fat < 30 % of total energy (E %), saturated fat < 10 E % and fiber ≥ 15 g/1000 kcal. The control group received general advice on the benefits of weight reduction, physical activity and healthy diet. The completeness of the food records was checked by the study nutritionist during each of the study visit. The mean daily nutrient intakes and food group/product intake were calculated with a dietary analysis program developed at the National Public Health Institute, Helsinki, Finland (1).

In the DPS the main end-point was diagnosis of diabetes defined by the WHO 1985 criteria (plasma fasting glucose ≥7.8 or 2-h glucose ≥11.1) to be confirmed by a repeated positive OGTT and verified by a physician. As explained in detail elsewhere (1, 2) after a median follow-up of four years as suggested by the independent endpoint committee the intervention phase of the study was discontinued. After the intervention (active study) period, the post-intervention follow-up was carried out with annual examinations. As previously reported (1), of the original 522 participants, 366 individuals free of T2D participated in the post-intervention follow-up study at least once. They were further followed until diabetes diagnosis, dropout or the end of 2009 (median total follow-up of nine years and time span of 13 years from baseline). Of these, 62 new cases of diabetes out of 200 in the intervention group and 68 out of 166 in the control group were diagnosed. During this period, 36 participants withdrew and ten died without a verified diabetes diagnosis (1).

*Calculations*

In the OGTT performed during follow-up visits starting from the middle of 1996, samples were also taken for 30 min insulin and glucose measurements. For this study, data for estimating insulin secretion were available for 350 subjects (n=187, lifestyles and n=163, control group) who had annual OGTTs with samples taken for 30 min insulin and glucose measurements. As surrogate index of the first/early-phase insulin secretion we used the disposition index30 (DI_30_), which was calculated as the product of the ratio of total insulin area under the curve (AUC) and total glucose AUC during the 0-30min OGTT multiplied by the Matsuda ISI, as previously (3-5). The DI_30_ annual values were averaged from the available yearly measurements. The measurements taken at the year of T2D diagnosis were not used for the calculation of the estimates and were not averaged to the values.

*References*

1. Lindström J, Peltonen M, Eriksson JG, Ilanne-Parikka P, Aunola S, Keinanen-Kiukaanniemi S, et al. Improved lifestyle and decreased diabetes risk over 13 years: long-term follow-up of the randomised Finnish Diabetes Prevention Study (DPS). Diabetologia. 2013 Feb;56(2):284-93.

2. Tuomilehto J, Lindström J, Eriksson JG, Valle TT, Hämäläinen H, Ilanne-Parikka P, et al. Prevention of type 2 diabetes mellitus by changes in lifestyle among subjects with impaired glucose tolerance. N Engl J Med. 2001 May 3;344(18):1343-50.

3. Stancáková A, Javorsky M, Kuulasmaa T, Haffner SM, Kuusisto J, Laakso M. Changes in insulin sensitivity and insulin release in relation to glycemia and glucose tolerance in 6,414 Finnish men. Diabetes. 2009 May;58(5):1212-21.

4. de Mello VD, Lindstrom J, Eriksson JG, Ilanne-Parikka P, Keinanen-Kiukaanniemi S, Pihlajamaki J, et al. Markers of cholesterol metabolism as biomarkers in predicting diabetes in the Finnish Diabetes Prevention Study. Nutr Metab Cardiovasc Dis. 2015 Mar 28;25(7):635-42.

5. de Mello VD, Paananen J, Lindstrom J, Lankinen MA, Shi L, Kuusisto J, et al. Indolepropionic acid and novel lipid metabolites are associated with a lower risk of type 2 diabetes in the Finnish Diabetes Prevention Study. Sci Rep. 2017 Apr 11;7:46337.

**1.2. Quantitation with HPLC-QQQ-MS/MS**

*Materials*

Calibration standard 3-indolepropionic acid (3-IPA) was purchased from Sigma-Aldrich (p.n. 220027, CAS 830-96-6) and internal standard indole-3-propionic-2,2-d2 acid (3-IPA-d2, IS) was purchased from C/D/N Isotopes Inc. (C/D/N Isotopes Inc., Pointe-Claire, Quebec, Canada, p.n. D 7686). The calibration standard and the internal standard were solubilized in 100 % methanol to get stock solutions. The working solutions were prepared by diluting the stock solutions in 50 % (v/v) methanol in water. Both the stock and working solutions were stored in -20 °C.

*Sample preparation*

Serum samples were stored in -80 °C. Samples were thawed and placed immediately on ice until sample preparation. Four hundred microliters of extraction solution composed of acetonitrile (ACN) including 50 ng/ml internal standard was dispensed to 96-well filter plates (Captiva ND Plate, 0.2 µm PP, Agilent Technologies, Santa Clara, CA, United States) and 100 microliters of vortexed serum/calibration standard working solution was added. The solution was mixed by pipetting up and down, filtrated by centrifugation (700 RCF, 10 °C, 5 min) and collected to 96-well plate (96 deep well plate natural, Thermo Scientific).

*HPLC-QQQ-MS/MS*

The HPLC system that was composed of Agilent 1200 Series Rapid Resolution LC System (Agilent Technologies) equipped with solvent degasser, a binary pump, a thermostated column compartment and a autosampler. The autosampler temperature was fixed at 10 °C. The chromatographic separation of the compounds was performed with the Zorbax Extend-C18, Rapid Resolution HT 2.1 x 50 mm 1.8-µm (Agilent Technologies, Santa Clara, CA, USA). The column temperature was set 60 °C. The elution gradient consisted of phases A (0.1 % formic acid in water) and B (0.1 % formic acid in MeOH). The elution initial gradient conditions were 20 % B, which was increased linearly to 95 % in 2.5 min, kept constant for 2 min and decreased to reach 20 % in 0.01 min. The column was equilibrated for 2.49 min with 20 % B before the next run. In chromatographic separation 5 µl of sample/standard was used and the flow rate was set 0.4 ml/min. The mass analyses were made with Agilent 6410 Triple Quad LC/MS and an electrospray source (Agilent Technologies). The drying gas (nitrogen) temperature was 300 °C, gas flow 8 l/min and capillary voltage 4000 V. The collision gas was argon. The quantitation was made using positive ion mode using multiple reaction monitoring (MRM). The monitored ion transitions were for 3-IPA 190.1->130 and 190.1->55 (CE 20 V) and for 3-IPA-d2 (IS) 192.1->130 and 192.1->57 (CE 20 V). The data was collected using MassHunter Workstation Acquisition software (Agilent Technologies).

*Method validation*

Calibration range of 25 - 5000 ng/ml was used. The calibration standards were injected in the beginning and in the end of the runs and calibration curves were generated using 1/x^2^ weighting. Criteria for the calibration standards used in calibration curve was 100 ± 15 % accuracy (relative error) of the nominal concentration.

Matrix effect of the method was studied using standard addition method. Four different serum samples were divided into three sub-samples and spiked with three concentrations of 3-IPA spiking solutions (+250 ng/ml, +500 ng/ml, +750 ng/ml). The samples were further extracted for HPLC-QQQ-MS/MS as in “*Sample preparation*”. The standard addition method was used to calculate 3-IPA concentrations in un-spiked serum. The concentrations were further compared to the concentrations achieved by internal standard method. For calculating the bias possibly caused by the serum itself following equation was used: bias% = 100(X−μ)/μ, where X is the concentration from standard addition method and µ is the concentration from internal standard method (Lehtonen *et al*. 2011).

Precision and recovery were determined by analyzing spiked serum samples. Pool of serum (QC1) was subdivided into three subsamples. Two of the subsamples were spiked with 3-IPA spiking solutions +250 ng/ml (QC1) or 750 ng/ml (QC3). Spiked and un-spiked samples were run as six replicates in three different days. Intra- and inter-day precision was calculated and presented as RSD for each concentration. The precision determined at each concentration should not exceed ±15 %. The recovery was calculated as in Lehtonen *et al.* (2011) using the spiked samples: recovery (%) = 100(S-U)/C, where S is the concentration of spiked sample, U represents concentration of un-spiked sample and C nominal concentration of the sample.

Stability of the IPA was evaluated by determining freezing-thawing and long term stability using serum pool that was divided into four, three of which were spiked with the analyte: STAB, STAB +125 ng/ml, STAB +500 ng/ml, STAB +2500 ng/ml) and froze in -80 °C. The freezing-thawing stability was determined after four freeze-thaw cycles. The long-term stability was evaluated by analyzing STAB samples stored in -80 °C 25 weeks.

**2. Results**

**2.2 Method validation for HPLC-QQQ-MS**

In order to evaluate matrix effect caused by the serum itself standard addition method was used. The systemic biases between the serum concentrations gained using standard addition method and internal standard method for four different serum samples were 0.3 %, 1.7 %, 1.9 % and 8.1 % (Figure S1).

The inter-run precision calculated and expressed as relative standard deviation (RSD) were for QC1 3.6 %, QC2 2.6 % and QC3 3.4 %. The intra-run precisions in three different days were 1.6-5.0 %, 1.6-3.4 % and 3.1-7.8 % for QC1, QC2 and QC3, respectively. Recovery was determined using the results gained for QC2 and QC3 from the runs evaluating the precision. Recovery in the three different runs was 95.5-98.5 % for QC2 and 88.8-94.9 % for QC3. Long term and freezing-thawing stability were defined for spiked serum samples. On the bases of the analyzes there was no significant degradation of the analyte resulting from the storing samples in -80 °C for 25 weeks or after four freezing-thawing cycles compared to the outset. For the stored samples analyte concentrations were 86.5. %-100.7 % and for the repeatedly freezed and thawed samples 92.6 %-108.1 %.

In conclusion, within a calibration range of 25-5000 ng/ml the HPLC-QQQ-MS/MS method for IPA, that was developed in this study, was precise, accurate and selective.

**2.3. Quantitative analysis of indolepropionic acid (IPA)**

Altogether, 415 serum samples were measured. The concentration of IPA was below the dynamic quantitation range for eleven samples (< 25 ng/ml) and above for one sample (> 5 000 ng/ml). Therefore, 403 samples (202 lifestyle intervention, 201 control) were included in the final analyses. IPA concentrations obtained from HPLC-QQQ-MS/MS correlated very well (r=0.99, p=9.1 x 10^-137^) with the IPA relative peak area obtained from the non-targeted metabolite profiling analysis (n=200) (2).

*References*

1. Lehtonen M, Storvik M, Malinen H, Hyytiä P, Lakso M, Auriola S, et al. Determination of endocannabinoids in nematodes and human brain tissue by liquid chromatography electrospray ionization tandem mass spectrometry. J Chromatogr B Analyt Technol Biomed Life Sci. 2011 Apr 1;879(11-12):677-94.

2. de Mello VD, Paananen J, Lindstrom J, Lankinen MA, Shi L, Kuusisto J, et al. Indolepropionic acid and novel lipid metabolites are associated with a lower risk of type 2 diabetes in the Finnish Diabetes Prevention Study. Sci Rep. 2017 Apr 11;7:46337.

**STable 1**. Serum IPA concentrations (ng/ml) according to rs7903146 and rs12255372 genotype of *TCF7L2* and study groups in participants of the Finnish Diabetes Prevention Study

| **rs7903146*** | Lifestyle | Control | **rs12255372**** | Lifestyle | Control |
| --- | --- | --- | --- | --- | --- |
| CC | 198 (130; 292) | 174 (109; 271) | GG | 208 (123; 303) | 172 (107; 263) |
| *n* | 113 | 122 | *n* | 121 | 128 |
| CT | 173 (104; 304) | 199 (85; 332) | GT | 174 (99; 285) | 205 (87; 341) |
| *n* | 71 | 59 | *n* | 62 | 56 |
| TT | 163 (121; 273) | 160 (107; 220) | TT | 190 (125; 269) | 146 (111; 174) |
| *n* | 8 | 11 | *n* | 9 | 8 |

* *P*=0.68 for the genotype effect in models adjusted for study group. *P*=0.43 for the genotype vs. study group interaction.

** *P*=0.88 for the genotype effect in models adjusted for study group. *P*=0.37 for the genotype vs. study group interaction.

**STable2.** Correlation coefficients (*r*) between the intakes of fat and types of fat reported by the participants and indolepropionic acid (IPA) serum concentrations at 1-year study

|  | IPA (ng/ml) | |
| --- | --- | --- |
|  | *r* | *P* |
| Fat intake, en% | -0.09 | 0.06* |
| g/d | 0.05 | 0.36 |
| SFA intake, en% | -0.11 | 0.03** |
| g/d | 0.00 | 0.95 |
| MUFA intake, en% | -0.10 | 0.05*** |
| g/d | 0.04 | 0.47 |
| PUFA intake, en% | -0.02 | 0.77 |
| g/d | 0.07 | 0.17 |

SFA: saturated fatty acids; MUFA: monounsaturated fatty acids; PUFA: polyunsaturated fatty acids

* *P*=0.71 for partial correlation adjusted for the intake of fiber

** *P*=0.69 for partial correlation adjusted for the intake of fiber

*** *P*=0.43 for partial correlation adjusted for the intake of fiber

**Figures**

**Figure S1.** The matrix effect was studied for four different serum samples by standard addition method. Three concentration of 3-IPA was spiked in to serum. Calibration curve was also prepared. The systemic biases for the serum 1, serum 2, serum 3 and serum 4 were 0.3 %, 8.1 %, 1.9 % and 1.7 %, respectively.
